# Cigarette and Nargila (Water Pipe) Use Among Israeli Arab High School Students: Prevalence and Determinants of Tobacco Smoking

**DOI:** 10.1100/tsw.2008.71

**Published:** 2008-05-22

**Authors:** Liat Korn, Racheli Magnezi

**Affiliations:** Department of Health Management, School of Health Science, Ariel University Center of Samaria, Ariel, Israel

**Keywords:** smoking, adolescents, nargila, religion, Arabs, Israel

## Abstract

Cigarette smoking is a popular habit among Arab Israelis. Over the past decade, smoking tobacco using nargila, a water pipe, has become a popular and accepted behavior among teenagers in Israel. Although the use of a water pipe (nargila) is an old habit among Middle Eastern adult males, its emergence among youth is a new finding. A representative sample of high school students in Tayibe, Israel is the subject of this survey. The sample represents data from 326 adolescents (boys 52.5% and girls 47.5%), ages 15–18, studying in one of the largest high schools in the Arab region of Israel. Our results show that a third of the sample smoked either cigarettes (36.2%) or nargila (37.1%). The gender difference among youths smoking cigarettes was 24.8% (48.0% for boys and 23.3% for girls), in contrast to 37.6% (55.0% for boys and 17.4% for girls) for nargila. There was a statistically significant correlation between cigarette and nargila smoking in populations where there is low religious inclination, increased parental smoking, and low student academic achievement. Students’ perceptions of low academic achievement (OR 4.51, *p* < 0.001), students’ mothers who smoke (OR 3.57, *p* < 0.001), and student's fathers who smoke (OR 2.75, *p* < 0.01) increase the youths’ chances of using nargila. Our conclusions are that smoking cigarettes and nargila are equally popular, and patterns of smoking cigarettes and nargila parallel each other. Causes that influence cigarette smoking also influence nargila smoking. Educational efforts are needed as a public health intervention.

## INTRODUCTION

Cigarette smoking is a major public health risk associated with various diseases and negative health outcomes[1]. A third of the world's male population smokes cigarettes[2]. In Israel, cigarette smoking (Knowledge Attitudes & Practice [KAP] survey) is more popular among the Arab Israeli population than the Jewish Israeli population[1]. Smoking prevalence among Arab Israeli women is low in contrast to Jewish Israeli woman (6.2 vs. 20.0%). Smoking prevalence is higher among all populations in Israel

(25.5%) compared to populations in the U.S. (20.9%)[3]. On the other hand, smoking prevalence in the Israeli, among both Israeli Arabs and Jews, is declining.

Heavy adult smokers start this habit at a young age[4]. Peer pressure and influence are strongest among adolescents compared to any other age group; therefore, it is important to intervene and set up programs among this age group and at this point of their lives.

The Health Behavior in School-aged Children (HBSC) study in Israel[5] showed a higher smoking prevalence among Arab Israeli boys (33.4%) in contrast to Jewish Israeli boys (26.8%) in 2006. Jewish Israeli girls had a higher smoking prevalence (19.1%) than Arab Israeli girls (10.2%). Arab Israeli boys smoked at least once a week more than Jewish Israeli boys (12.6 vs. 6.8%), whereas Jewish Israeli girls smoked more than Arab Israeli girls (4.1 vs. 2.6%).

Theories that explain health-risk behaviors noted a clustering effect with risk behaviors and they did not remain as isolated risk behaviors[6,7,8]. The risk behavior of smoking was more commonly associated with other risk behaviors, such as problematic alcohol use, illicit drug use, or violent behavior[9]. Kandel explained drug use among adolescents in the "stage development theory for drug use" or "Gateway Theory"[10,11,12]. According to this theory, there is a progression in the use of drugs from light legal substances to more dangerous illegal drugs. The progression starts with smoking cigarettes and progresses to the use of hard illicit drugs. Alcohol consumption and cigarette smoking may act as "gateways" to the possible use of hard drugs. These observations lead us to believe that Arab Israeli boys may be in a high-risk group that warrants intervention.

Nargila smoking has become a popular and common new risk behavior among Israeli adolescent school-aged children, Jews and Arabs[13,14]. Nargila smoking used to be a cultural activity limited to adult Middle Eastern males, however, several years ago, the Israeli youth adopted this social activity ("risk behavior"). Children ages 11 years and older use nargila in Israel. Over a 3–5 year period, the prevalence rate of nargila smoking among Israeli teens (under the age of 18) rose from 0 to 40%[15].

Preliminary studies showed nargila smoking as a popular habit in Arab countries in the Middle East. In Lebanon, 15% of the population use nargila[16]; 27% of pregnant women in Beirut smoke nargila[17]; and among adolescents in Beirut, 9% report daily use, whereas 39% report using nargila occasionally[18]. In Egypt, 30% of customers in a coffee shop admitted having a nargila device in their homes and 94% have a friend who also smokes tobacco using nargila[19]. In the last few years, Syria reported a rise in nargila smoking[20]. Nargila smoking is also popular in Sudan and Tunisia[21,22]. In Israel, 2002 HBSC data using a representative sample revealed that 30.5% of Arab Israeli students have tried using nargila at least once and 9.3% use nargila regularly, daily, and once a week[15].

Worldwide studies showed nargila smoking as a health-risk behavior[23,24]. Multiple studies linked nargila smoking with lung disease; cancer of lung, mouth, and cheek; hypertension; chronic respiratory disease, and ulcer disease[25,26]. An associated increase in the incidence of cardiovascular disease (pulse, blood pressure, carbon dioxide levels in the blood) has also been found[27] and there is danger to the health of unborn babies[28]. Routine and repetitive use of nargila can cause a long-term decrease in blood oxygen concentrations[29]. These studies showed nargila smoking to be as equally dangerous to health as cigarette smoking.

Studies have associated smoking with peer influence and socioeconomic and environmental influence. Socioeconomic status influences many health indicators. A Scottish study[30] showed an association of social position, income, housing tenure, and car access to health. Studies also determined an association of socioeconomic status with lower education levels and increased smoking. There is a higher incidence of smoking among nonacademic in contrast to academic individuals[1]. Among academic vs. nonacademic Arab males, smoking prevalence varies from 41 to 51.5%. Among Arab Israeli woman, academic vs. nonacademic prevalence also shows a similar difference, 3.0 vs. 8.9%. Acik et al. showed an association between religious inclination and health-risk behaviors[31]. One study from the Netherlands showed that religious adolescents had lower smoking incidence in contrast to nonreligious adolescents[32].

Difficult relationships between adults and youngsters carry a higher likelihood of smoking, use of cannabis, and drinking alcohol[33], but parents can also influence their adolescents by engaging in risk behaviors in other ways. Children who live with parents that smoke have a greater likelihood of having asthma. In addition, if the child is not an asthmatic, they still have a greater likelihood of smoking if they come from a smoking household[34]. One study showed a higher smoking prevalence and higher recognition of tobacco brands among children from smoking households[35].

School perceptions showed significant association with risk behavior. Many studies showed strong associations between risk behaviors, such as smoking, and negative school perceptions[36]. Younger students with positive school perceptions had significantly less risk behaviors (such as smoking and drinking). Adolescents with higher grades in school showed lower smoking prevalence[37,38].

There are two purposes for this study: to examine prevalence rates of cigarette and nargila smoking among the Arab Israeli teenagers, and to explore social determinants and their influence on the likelihood of a teenager developing a smoking habit. This research addresses a previously unexplored subpopulation, the Arab Israeli adolescent. Determinants that influence nargila use in this population are also not available in literature.

## METHODS

Our population and sample were 10th, 11th and 12th grade high school adolescents, ages about 15–18, studying at the largest Arab high school in Israel. Many adolescents attend this high school from the city of Tayibe and surrounding villages. The survey included all students who were present in the sampled classroom on the day the researchers gave the questionnaire. The sample size that completed the questionnaires included 326 students (boys 52.5% and girls 47.5%). This sample size represented 30% of all high school students at the school. The sample represented 105 students (32.2%) from the 10th grade, 113 students (34.7%) from the 11th grade, and 108 students (33.1%) from the 12th grade.

The survey used a classroom self-administered anonymous questionnaire developed and tested over the years as part of the international HBSC scientific collaboration. The surveyors added a few measures of particular interest unique to this population. The forms contained specific questions about nargila use. Published copies of detailed descriptions of the method of the international[39,40] and Israeli[15] HBSC, including information of questionnaire development and method, were available. Researchers used the SPSS program for the analysis of the collected data collected.

There were 14 questions in the form. The following are groupings of the questions:

- Four questions on demographics (age, gender, religion, and socioeconomic status)
- One question on perception of academic achievement
- Two questions about parental smoking habit
- Four questions on students cigarette smoking habit
- Three questions on students nargila smoking habit

Use of cigarettes and nargila were the dependent variables in this study. The two measured behaviors associated with cigarette smoking were "one time cigarette use during their lifetime" and "current frequency of cigarette smoking". The former answers the question: "Did you ever smoke a cigarette?" (1 = yes, 0 = no), whereas the latter answers the question: "How often do you smoke cigarettes?" Measured behaviors associated with nargila were "one time use of nargila pipe in their lifetime" and "current frequency of smoking nargila". The former answers the question: "Did you ever smoke tobacco using nargila pipe?" (1 = yes, 0 = no) and the latter answers the question: "How often do you smoke nargila?" The students answered the question as follows: 1 = smoke at least once a week, and 0 = do not smoke or smoke less than once a week.

Independent variables included demographics (gender, grade, religious inclination, and socioeconomic status), parental smoking, and school perceptions:

- **Parental smoking** — The questionnaire asked "if either their mother or father smokes". For each person (mother and father), the student answered with a number from 1 to 4. Each number represented an answer as follows: 1 (smokes every day), 2 (smokes sometimes), 3 (does not smoke), and 4 (do not know). The positive answer to the issue of a "smoking parent" was answer number 1 only.
- **School achievement** — Students answered the question: "How would you estimate your school achievement?" with the following assigned numbers: 1 (very good), 2 (average), 3 (below average).

## RESULTS

Table 1 presents percent distribution and confidence intervals of variables by gender. Fig. 1 is a bar graph showing the percentage of students that smokes cigarettes and nargila by religious preference. The data show that cigarette smoking and nargila smoking are more common (males and females) among those with secular backgrounds (78.5 and 75.0%, respectively) in contrast to conservative (35.9 and 38.5%, respectively) or religious backgrounds (27.4 and 25.9%, respectively). Frequencies for both cigarettes and nargila of "one time smokers" or "frequent smokers" are higher among secular than conservative or religious students. The percent difference between students that smoke is greater between secular and conservative groups vs. conservative and religious groups.

**TABLE 1**
**Distribution of Variables by Gender**

| Measure (%) | All Students | Gender | |
|---|---|---|---|
| | | Boys | Girls |
| Dependant variables | | | |
| Students that have smoked cigarettes | 36.2 (±0.48) | 48.0 (±0.50) | 23.2 (±0.42) |
| Students that smoke cigarettes once a week or more | 15.8 (±0.36) | 22.5 (±0.41) | 8.5 (±0.27) |
| Students that have smoked nargila | 37.1 (±0.48) | 55.0 (±0.49) | 17.4 (±0.38) |
| Students that smoke nargila once a week or more | 9.8 (±0.29) | 15.8 (±0.36) | 3.2 (±0.17) |
| Nondependant variables | | | |
| Students that are from an average or below average socioeconomic status | 39.0 (±0.48) | 47.6 (±0.48) | 29.1 (±0.49) |
| Students that are from a secular background | 8.6 (±0.28) | 12.9 (±0.33) | 3.9 (±0.19) |
| Students that have an average to below average perception of their academic achievement | 39.3 (±0.48) | 40.9 (±0.49) | 37.4 (±0.48) |
| Students whose mothers smoke | 28.1 (±0.45) | 24.0 (±0.42) | 32.7 (±0.47) |
| Students whose fathers smoke | 67.3 (±0.46) | 62.0 (±0.48) | 73.2 (±0.44) |
| Total | 326 | 171 | 155 |

Fig. 2 represents the percentage of students that smoke cigarettes and nargila, their religious background, as well as individual parent smoking habit. Students whose mothers smoke and are from secular families smoke the greatest percentage of cigarettes or nargila. Results showed a higher percentage of children that smoke if their mothers smoke in contrast to if their fathers smoke. This is especially true in families with a secular background. In secular families where mothers smoke, there is a greater frequency of children who smoke nargila instead of cigarettes.

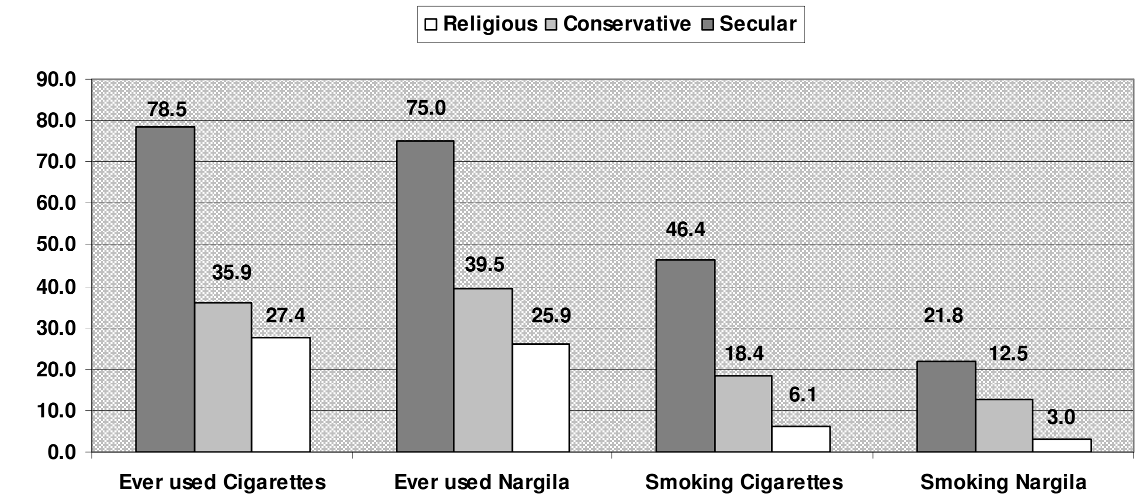

**FIGURE 1.** Bar graph representation of the percentage of students that smoke cigarettes and nargila by religious conviction; $p < 0.001$ for the four time reference, n = 326.

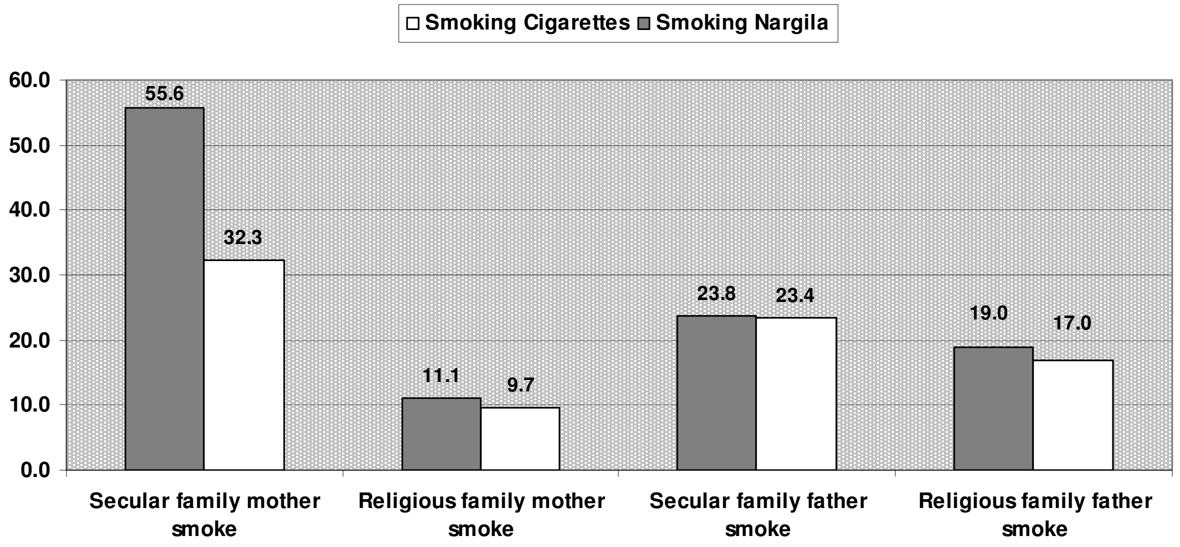

**FIGURE 2.** Percentage of cigarette and nargila smokers by religion and whose parents smoke; $p < 0.05$ for the two time reference, n = 326.

Table 2 represents the percentage of students that smoke cigarettes and nargila, their perception of their academic achievement, and their gender. The data show that in both genders there is an inverse association between smoking and perceived academic achievement. The frequency of boys that smoke nargila and have a high perception of their academic performance is lower (7.4%) compared with the frequency of boys that smoke nargila and have low perception of their academic performance (92.6%).

Table 3 is a logistic regression predicting the probability of smoking both cigarettes and nargila. There are three groups: students smoking once only, smoking fewer than once a week, smoking at least once a week. The table also shows the odds ratio (OR) for smoking cigarettes and nargila using the following predicting variables: gender, socioeconomic status, religion, academic achievement, mother who smokes, and father who smokes.

**TABLE 2**
**Percent Distribution of Cigarette and Nargila Smoking by Academic Achievement and Gender***

| | Girls | | Boys | |
|---|---|---|---|---|
| | Smoke | Do Not Smoke | Smoke | Do Not Smoke |
| Cigarettes | | | | |
| High academic achievement | 23.1 (n = 3) | 65.7 (n = 92) | 21.1 (n = 8) | 69.5 (n = 91) |
| Low academic achievement | 76.9 (n = 10) | 34.3 (n = 48) | 78.9 (n = 30) | 30.5 (n = 40) |
| Total | 100.0 (n = 13) | 100.0 (n = 140) | 100.0 (n = 38) | 100.0 (n = 131) |
| Nargila | | | | |
| High academic achievement | 20.0 (n = 1) | 64.0 (n = 96) | 7.4 (n = 2) | 68.8 (n = 99) |
| Low academic achievement | 80.0 (n = 4) | 36.0 (n = 54) | 92.6 (n = 25) | 31.3 (n = 45) |
| Total | 100.0 (n = 5) | 100.0 (n = 150) | 100.0 (n = 27) | 100.0 (n = 144) |

* $p < 0.05$ for the two time reference, n = 326–322.

**TABLE 3**
**Odds Ratio of Nargila and Cigarette Use (Predicting Variables): Logistic Regression Data**

| Variables | Cigarettes | | | Nargila | | |
|---|---|---|---|---|---|---|
| | Never Used | Used at Least Once in Lifetime, but Less than Once a Week | Used at Least Once a Week | Never Used | Used at Least Once in Lifetime, but Less than Once a Week | Used at Least Once a Week |
| Gender | 1.00 | 7.20*** | 4.63*** | 1.00 | 10.66*** | 5.96*** |
| SES | 1.00 | 0.97 | 0.19** | 1.00 | 0.61 | 1.01 |
| Religion | 1.00 | 1.93 | 2.00* | 1.00 | 1.61 | 1.14 |
| Academic achievement | 1.00 | 5.74*** | 11.33*** | 1.00 | 4.51*** | 25.97*** |
| Mother smokes | 1.00 | 11.19*** | 4.00** | 1.00 | 3.57*** | 0.59 |
| Father smokes | 1.00 | 1.73 | 6.08** | 1.00 | 2.75** | 0.60 |
| Adj $R^2$ | — | 50.8% | 43.3% | — | 44.0% | 35.7% |
| N | 208 | 67 | 51 | 205 | 89 | 32 |

The significant value: ***$p < 0.001$, **$p < 0.01$, *$p < 0.05$.

Findings suggest 50% explained variance for cigarette smoking at least once, but no more than once, a week; 43.3% explained variance of "weekly" use of cigarettes. It also shows a 44% explained variance of smoking using nargila at least once, but fewer than once, a week; 35.7% explained variance of "weekly" use of nargila. There is better prediction of variables in the moderate use of tobacco (less than once a week) for both cigarettes and nargila, than in the more intense use of these products (at least once a week).

Results suggest that students have the highest probability of smoking cigarettes (less than once a week) if their mothers smoke (OR: 11.19, $p < 0.001$). A boy, having a perception of low academic achievement, raises his probability of smoking (OR: 7.20 and 5.74, respectively, $p < 0.001$). There was little association with students' probability of trying a cigarette with the other variables of socioeconomic status, religious background, and students' fathers that smoke. However, the probability of "weekly use of cigarettes" increased with all variables. A boy who has a low perception of his academic achievement and both parents who are smokers has a higher probability of smoking regularly. A child has a greater risk of becoming a moderate cigarette smoker if their mother is a smoker. Adolescents have a greater risk of smoking cigarettes at least once a week if their fathers are smokers.

The data reveal that male gender is the most significant predictor for trying to smoke nargila at least once (OR: 10.66, $p < 0.001$). A perception of low academic achievement statistically significantly increases the probability of smoking nargila (OR: 4.51, $p < 0.001$). If a student's mother smokes, the odds ratio increases to 3.57 ($p < 0.001$), whereas if a student's father smokes, the odds ratio is 2.75 ($p < 0.01$). A low self-perception of academic achievement is the most significant variable that increases the probability of smoking nargila regularly (OR: 25.97, $p < 0.001$). The next significant variable is being a male (OR: 5.96, $p < 0.001$). The data suggest that in the Arab Israeli population, perception of low academic achievement and a smoking household are two strong influences in increasing the probability of smoking by the youngster. Children with perceptions of low academic achievement have a greater probability of smoking (both cigarettes and nargila) at least once a week. This variable, therefore, is an important predictor in determining smoking frequency in intense use.

## DISCUSSION

There were two purposes for this study. First, to examine the prevalence of cigarette and nargila smoking in Arab Israeli teenagers, and second, to explore social determinants and their influence on the probability of a teenager trying and developing a smoking habit. This study examined the smoking prevalence among Arab Israeli teenagers of cigarettes and nargila. It looked at determinants of smoking, such as religious background, parental smoking history, and student perception of school achievements. The Arab Israeli sample used in this research showed outcomes similar to other previously published studies on smoking tobacco as cigarettes and using nargila. The uniqueness of this study is explaining smoking prevalence (cigarettes and nargila) in the Arab Israeli population and gaining insights into cultural and social influences on this risk behavior.

Our research showed that lifetime prevalence of nargila use among Arab Israeli high school children was 50% among boys and 25% among girls. The difference in gender is higher in this population compared to previous findings among the Jewish Israeli population. Findings may suggest a more conservative and male-dominated society among the Arab Israeli in contrast to the Jewish Israeli society. In the former society, smoking tobacco using nargila is more of a "masculine behavior". Also, girls that are more discontent with social restrictions use nargila pipe for smoking tobacco in this society. This may explain the greater variance between male and female among nargila users compared with cigarette smokers.

This study showed that a higher percentage of students from a secular and conservative religious background, in contrast to a religious background, more often tried cigarette smoking and nargila. There is a greater number of practicing religious people among the Arab Israeli in contrast to the Jewish Israeli population. Previously published studies in this population did not show a strong association between smoking and religious inclination. This study showed religious background to be an important determinant that predicts probability of smoking among Arab Israeli adolescents. In this population, there is a greater disparity in the percentage of smokers between the religious and secular beliefs.

In secular families where mothers smoke, there is a greater frequency of children who smoke using nargila instead of cigarettes. There is a belief in the general population that nargila smoking is less dangerous to health compared with cigarette smoking. Nargila use is a new gateway for tobacco consumption. Additionally, mothers who smoke have a greater impact on the child's smoking habit, especially on the daughters more than the sons. These findings support the theory of gender socialization.

The data also showed an inverse association between smoking and academic achievement. This association is even stronger with nargila than cigarette smoking. Previous studies consider nargila use as a low-risk "introductory" behavior in the gamut of "substance use". In the past, nargila use was low in the epidemiological pyramid, among many teen populations, and levels of risk behavior. This study showed that in the Arab Israeli population, nargila smoking is more strongly tied to low academic achievement than cigarette smoking. One possible explanation could be that nargila smoking consumes a greater deal of time than does cigarette smoking, therefore, daily use of nargila detracts from time available for academics and, hence, poor academic achievement. In the Arab Israeli student population, nargila smoking is a stronger health-risk behavior than cigarette smoking.

## CONCLUSIONS

This study showed that smoking tobacco using cigarettes or nargila is equally popular among Arab Israeli teenagers. Patterns and factors that influence both these smoking methods parallel each other. Future studies should further explore the impact of religious background on nargila and cigarette smoking. Realizing this important health-risk behavior, nargila smoking should be more carefully and rigorously supervised by national high-risk behavior institutions. Implementation of health education campaigns dealing with this new and rampantly spreading behavior is essential.

## ACKNOWLEDGMENTS

The authors thank Ms. Nashf Riham and Ms. Mansur Afaf, and especially Prof. Gabriel Amitai, for their contributions in the writing of the article.
